# Breadmaking Performance of Elite Einkorn (*Triticum monococcum* L. subsp. *monococcum*) Lines: Evaluation of Flour, Dough and Bread Characteristics

**DOI:** 10.3390/foods12081610

**Published:** 2023-04-10

**Authors:** Andrea Brandolini, Mara Lucisano, Manuela Mariotti, Lorenzo Estivi, Alyssa Hidalgo

**Affiliations:** 1 Consiglio per la Ricerca in Agricoltura e l’Analisi dell’Economia Agraria—Unità di Ricerca per la Zootecnia e l’Acquacoltura (CREA-ZA), Via Piacenza 29, 26900 Lodi, Italy; 2 Dipartimento di Scienze per gli Alimenti, la Nutrizione e l’Ambiente (DeFENS), Università degli Studi di Milano, Via G. Celoria 2, 20133 Milan, Italy; mara.lucisano@unimi.it (M.L.); mariotti.manu@gmail.com (M.M.); lorenzo.estivi@unimi.it (L.E.); alyssa.hidalgovidal@unimi.it (A.H.)

**Keywords:** colour, farinograph, rheofermentograph, viscoelastic behaviour, bread shelf life, crumb porosity

## Abstract

Einkorn flour, rich in proteins, carotenoids, and other antioxidants, generally has poor breadmaking value. In this research, the composition and technological characteristics of the flours and breads of two elite einkorns (Monlis and ID331) and a bread wheat (Blasco), cropped in four different environments, were evaluated. The einkorns confirmed better flour composition than bread wheat for proteins (on average, 16.5 vs. 10.5 g/100 g), soluble pentosans (1.03 vs. 0.85 g/100 g), and yellow pigment (10.0 vs. 1.0 mg/kg). Technologically, they had better SDS sedimentation values (89 vs. 66 mL), lower farinographic water absorption (52.6 vs. 58.8%), and a similar development time, stability, and degree of softening. Viscoelasticity tests showed lower storage and loss moduli and more prevalent elastic behaviour for Blasco, while rheofermentographic tests showed an anticipated development time (120.8 vs. 175.0 min), higher maximum height (73.0 vs. 63.0 mm), and superior retention coefficient (99.1 vs. 88.7%), but a lower CO_2_ total (1152 vs. 1713 mL) for einkorn doughs. Einkorn breads were bigger than the control (736 vs. 671 cm^3^); crumb pores percentage was similar, but medium-size pores were scarcer. Finally, a 52-h shelf-life trial demonstrated that einkorn bread had a softer texture, maintained for a longer time, and a slower retrogradation than the control. Therefore, choice of appropriate varieties and process optimisation allows the production of excellent einkorn breads with a superior nutritional value and longer shelf life.

## 1. Introduction

Einkorn (*Triticum monococcum* L. subsp. *monococcum*), a diploid hulled wheat (2 *n* = 2 x = 14) with high protein [1,2,3], lutein, and antioxidants content [4,5], is considered a cereal with a poor baking attitude and a dough characterized by excessive stickiness. The farinographic tests often show evidence of scarce stability and a high degree of softening, while breadmaking yields small volume loaves, due to reduced leavening and easy collapse of the dough [6,7]. However, a great variation for breadmaking quality exists within the einkorn gene pool, and selected ecotypes with high SDS sedimentation values (>70 mL), good farinographic stability (360–720 s), and a limited degree of softening (20–50 UB) yielding breads with volumes similar or even higher than wheat breads have been identified [6,7,8]. Additionally, because their doughs present poor tolerance to mechanical processing and prolonged fermentation, gentle processing at a low speed and for a short duration (3–4 min) have been proposed to improve loaves’ volume [9]. The use of sourdough fermentation has also been suggested to improve texture, volume, and shelf life of einkorn bread [10]. Crust shape and colour are similar to those of wheat loaves, but the crumb shows an enticing yellow tinge [11] for the high carotenoids content [5]. Furthermore, the lower endogenous enzymatic activity [12,13] allows for better preservation of antioxidants during storage [14,15] and processing [16,17], and limits heat damage [18], thus safeguarding the favourable nutritional characteristics of einkorn-derived products.

The potential health benefits of einkorn foods have been the subject of recent investigations. Although einkorn, like other wheats, such as barley and rye, is not suitable for consumption by people with celiac disease [19,20,21], einkorn may be better suited than other *Triticum* species for patients with chronic dysmetabolic diseases. For example, einkorn bread consumption leads to more favorable metabolic responses and greater satiety compared with standard wheat breads [22]. Investigating the effects of einkorn bread on the intestinal physiology and metabolism of pigs, Barone et al. [23] observed a lower postprandial insulin rise after einkorn consumption compared with bread wheat consumption; furthermore, the intestinal ecosystem was enriched in health-promoting bacteria. Einkorn’s anti-inflammatory effects were also recorded in cultured cells [24].

Although einkorn breads are often characterized by an inferior technological quality compared with wheat breads, their enticing taste, aroma, attractive color, and health-promoting properties suggest that they may be a worthy addition to the increasing assortment of available products. Therefore, the aim of this research was to evaluate several breadmaking quality facets of two elite einkorn wheats (ID331 and Monlis) and, as control, of one bread wheat cultivar (Blasco); the two einkorn accessions were selected because in previous unpublished tests repeatedly showed the best breadmaking attitude. Hence, all-around information about the breadmaking properties of these two elite einkorns will allow the baking industry to develop new leavened products through adopting the most appropriate processing approaches. To this end, the composition and technological and rheological properties of their flours, doughs, and breads were assessed throughout the breadmaking process; furthermore, the shelf life of the bread loaves was investigated.

## 2. Materials and Methods

### 2.1. Materials

Two breadmaking-quality einkorns (ID331 and Monlis) and a breadmaking-quality bread wheat (cv. Blasco) were cropped in four different environments, two under conventional management (Sant’Angelo Lodigiano, LO, and Montelibretti, Rome, labelled as SAL and ROMA) and two under organic management (Sant’Angelo Lodigiano, LO, and Leno, BS, i.e., SALbio and LENObio). Some relevant agronomic parameters of the four environments are summarized in Table S1. The accessions were planted in 10 m^2^ plots according to a Randomized Complete Block Design with three replications. For weed control, the herbicide Ariane II (Clopiralid 1.8% + Fluroxypyr 3.6% + MCPA 18.2%; Dow AgroSciences, Milan, Italy) was applied just before heading to the conventional management plots, while the organic trials were manually weeded. After machine harvesting, by mid-July (Table S1), the Monlis and ID331 kernels were de-hulled with an Otake FC4S thresher (Satake, Japan), which is a step that was not necessary for the free-threshing bread wheat Blasco. All seeds were stored at 5 °C until further processing.

### 2.2. Methods

#### 2.2.1. Kernels

Kernel moisture was determined by a GAC2000 moisture analyzer (Dickey–John, Auburn, IL, USA) and corrected to 15% for the einkorns and 16% for Blasco (harder texture) by overnight tempering. Afterwards, the hectoliter weight (kg/hL) was determined with a GAC2000 instrument (Dickey–John, Auburn, IL, USA). The seeds were milled with a Bona–GBR laboratory mill (Bona, Monza, Italy), which separates flour from bran and germ, and the milling yield (% flour/kernels *w*/*w*) was computed.

#### 2.2.2. Flour and Dough

Flour particles’ size was determined by sifting 100 g of flour for 5 min through a sieve with a 125 μm mesh; the ≥125 vs. <125 ratio was computed. Flour characteristics were then assessed according to the following methods: moisture (AACC 44-15.02 [25]), ash content (AACC 08-03.01 [25]), protein content (N × 5.7; AACC 46-10.01 [25]), yellow pigment (AACC 14-50.01 [25]), dry gluten content (AACC 38-12.02 [25]) with a Glutomatic (Perten, Hägersten, Sweden), Falling number (AACC 56-81.03 [25]) with a 1550 Falling Number (Perten, Hägersten, Sweden), SDS sedimentation test (a breadmaking attitude predictor [26]), starch and amylose contents and α-amylase activity with Megazyme Assay Kits (Megazyme International Ireland Inc., Bray, Ireland), and pentosan and soluble pentosan contents (colorimetric method) [27]. Dough mixing properties were evaluated with a Brabender farinograph (Brabender OHG, Duisburg, Germany) using a 50 g mixer according to the ICC method 115-D [28]. Briefly, after adjusting the maximum consistency of the dough to a fixed value (500 Brabender Units, BU) by altering the quantity of water added, the test was run for 12 min. The parameters recorded were water absorption (amount of water added to set the curve at 500 BU, expressed as percentage of flour at 14% moisture), development time (time in minutes between the origin of the curve and its maximum value i.e., 500 BU), stability time (difference in minutes between the time to the maximum and time when the top of the curve falls below 500 line), and degree of softening (difference in BU between the maximum value and the value at the end of the test).

Dough leavening properties were assessed by rheofermentographic tests and were performed with a Chopin F3 Rheofermentometer (Chopin SA, Villeneuve-La-Garenne, France), according to [29]. The indices recorded from the curves were Hm (mm; maximum development of the dough), h (mm; height of the dough at the end of the test), CO_2_ total (mL; total gas production during the test), CO_2_ loss (mL; total gas loss during the test), CO_2_ retained (mL; gas retained by the dough during the test), and retention coefficient (% CO_2_ retained/CO_2_ total).

To better understand the fundamental properties of the doughs, rheological analyses were carried out according to [30] with a Physica MCR300 Rheometer, supported by US200 v. 2.5 software (PHYSICA Messtechnic GmbH, Ostfildern, Germany). Measurements were carried out at 25 °C using a corrugated parallel plate system (diametre: 2.5 cm) at a gap of 2 mm and a special humidity cover (H-PTD 150) to prevent moisture losses. Frequency sweep tests were performed over the range 0.1–50 Hz at 0.1% strain on doughs prepared at the same consistency (500 UB) after a resting period of 30 min to equilibrate stresses. The selected 0.1% strain value was obtained from preliminary amplitude sweep tests performed in the range of 0.01–100% strain, at a constant frequency of 1 Hz, to determine the linear viscoelastic region (LVR) of the sample. Each test was performed at least three times. Data were analyzed with US200/32 v. 2.50 rheometer software (PHYSICA Messtechnic GmbH, Ostfildern, Germany) and the value of storage modulus (G′, Pa) and loss modulus (G″, Pa) at 1 Hz and tanδ (ratio between G″ and G′) were computed.

#### 2.2.3. Bread

Two breads per accession were produced according to method 10-10 B [25] with minor modifications [6]. Bread weight (g) was determined with a LP5200P balance (Sartorius AG, Göttingen, Germany), bread volume (cm^3^) by rapeseed displacement (method 10-05.01 [25]), and bread height (mm) with a caliper; specific volume (cm^3^/g) was computed as volume-to-weight ratio.

Bread quality changes during storage were monitored on eight breads per accession, prepared only from SAL trial flours. Two breads were immediately analyzed, while the others were packaged in food paper bags and stored at 25 °C and 60% relative humidity in an HC 0020 air-conditioned cell (Heraeus, Hanau, Germany). Four times (t_0_: 0 h, t_1_: 24 h, t_2_: 30 h, t_3_: 52 h), two bread loaves of each accession were weighed to evaluate weight decrease during storage, then they were transversely cut to obtain three uniform 25-mm-thick slices. Crumb moisture was assessed on two slices while water activity (*a_w_*) analysis was performed on the third central slice with an AQUALAB apparatus (Decagon Devices Inc., Pullman, DC, USA).

Crust color and crumb color of the three slices of each sample at t_0_ were assessed in the CIELAB space using a Minolta Chroma Meter CR 210 (Minolta Camera Co., Osaka, Japan) with a standard illuminator C. Additionally, images were captured using a flatbed scanner (Scanjet 6300c; Hewlett Packard, Palo Alto, CA, USA) in 256 grey level at 300 dots per inch and were processed using a dedicated software (Image Pro-Plus 4.5.1.29, Media Cybernetics Inc., Rockville, MD, USA). The parameters evaluated were density red (R), density green (G), density blue (B), and density mean. At the same time, crumb porosity was determined by assessing the number, area, diameter, and shape of the pores, their size distribution (three categories: C1: 0.1–1 mm^2^, C2: 1–5 mm^2^, C3: >5 mm^2^), and % of pore area.

Crumb texture characteristics were assessed by Texture Profile Analysis on the three central slices from each sample at each storage time with an HD.plus Texture Analyzer TA double-column dynamometer (Stable Micro System, Godalming, UK) connected to a Texture Exponent 32 recording system version 4.0.8.0 (Stable Micro System, Godalming, UK). The operating conditions adopted were: load cell 500 N, crossbar speed 2 mm/s, compression plate diameter 36 mm, sample compression up to 40% thickness, and 25 s waiting time between 1st and 2nd compression cycles. The parameters derived from the Force/Time curve were: young modulus (N/mm^2^), hardness (N), springiness or elasticity (adimensional), cohesiveness (adimensional), and chewiness (i.e., hardness × cohesiveness × springiness, N).

#### 2.2.4. Statistical Analysis

A two-way analysis of variance (ANOVA) was carried out for most parameters, using environment and genotype, or genotype and storage time, as factors. A one-way ANOVA was performed on the results of the color and porosity parameters, assessed on the breads prepared from the SAL flours. When significant differences (*p* ≤ 0.05) were found, Fisher’s lowest significant difference (LSD) was computed. Both ANOVA and LSD testing were performed with the software STATGRAPHICS plus v.4 (STATPOINT Technologies Inc., The Plains, VA, USA). Means and standard errors were determined using the software Excel^®^ (Microsoft Corporation, Redmond, DC, USA).

## 3. Results

### 3.1. Kernels

**Figure 1:**
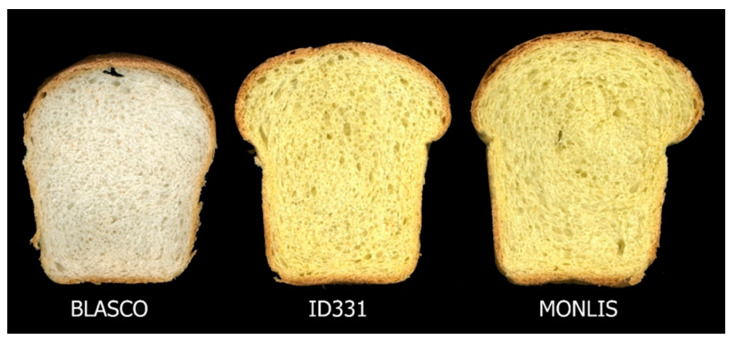
Bread slices prepared from flours of bread wheat Blasco, einkorn ID331, and einkorn Monlis.

Figure 1 reports the values of hectoliter weight, flour yield, and proportion of flour <125 μm, while their behavior in the four locations is depicted in Figure S1. The results in the different locations (Figure S1) suggest that the hectoliter weight of the bread wheat was generally higher than that of the two einkorns, while the flour yield and the percentage of flour particles <125 μm were higher in the einkorns than in the bread wheat Blasco. It is interesting to note that an elevated hectoliter weight, besides indicating healthy caryopses with a compact endosperm typical of “hard” type wheats, such as Blasco, usually forecasts high flour yields [31]. However, despite the smaller seeds and consequent superior teguments proportion [2], as mentioned above, the flour yield of both einkorns seems superior to that of the bread wheat Blasco. This peculiar result was also spotted by Borghi et al. [6] in their survey of 25 einkorns and by Corbellini et al. [7] in their study of 24 einkorns, whose flour yields ranged between 53.0 and 64.4% and were similar to the soft wheat Veronese (59.0%), but were superior to the hard wheat Pandas (50.0–52.6%). The higher extraction rate may be linked to the extra-soft texture of einkorn kernels [1,7], which makes them more easily grindable, and originates a very fine flour [6,32,33]. In this study, indeed, the percentage of einkorn flour particles smaller than 125 μm was, on average, 86.0 g/100 g (in the range of the 84.9–91.7 g/100 g reported by Borghi et al. [6]), while it was 57.8 g/100 g for Blasco. Probably during milling the harder bread wheat kernels broke into larger fragments, thus originating, in comparison to einkorn, a coarser flour. No major differences between ID331 and Monlis were observed.

### 3.2. Flour and Dough

A two-way ANOVA (Table S3) highlighted the existence of significant differences among accessions and among environments, as well as their interactions for moisture, ash, protein, total starch, amylose, soluble pentosans, total pentosans, and yellow pigment content. In all instances, the genotype was the most relevant factor. The results of the four environments, not discussed in this article, are summarized in Table S3.

The ash content of Blasco (Table 1) was significantly lower (0.58 g/100 g DM) than that of the two einkorns (on average, 0.63 g/100 g DM), as evidenced also by other authors [1,32,34]. Additionally, Blasco was less rich in protein and richer in starch than the two T. monococcum accessions.

Einkorn is well known for its high protein levels [1,6,7,32,33,35], which in part is due to genetic factors and in part to its smaller kernels having an inferior endosperm-to-external layers ratio and hence, a superior incidence of the protein-rich aleuronic layer [2]. Nevertheless, despite the considerable difference in protein concentration, the amino acid composition of all wheats is similar, with lysine representing the main limiting factor [36,37,38].

Conversely, the plump Blasco kernels were richer in starch than the smaller, protein-rich einkorn seeds (79.1 vs. 69.0 g/100 g). However, the amylose proportion of total starch was similar between Monlis and Blasco, and was only slightly inferior in ID331 (Table 1). On the other hand, Blasco had similar pentosan and inferior soluble pentosan contents than the einkorns (2.52 vs. 2.57 g/100 g and 0.82 vs. 1.03 g/100 g, respectively). The total pentosan concentration of Blasco was within the variation (2.0–3.0 g/100 g) described for bread wheat [39,40], while the soluble pentosan content fell into the range (0.49–1.23 g/100 g) observed in *T. aestivum* [27,41,42]. To the best of our knowledge, no information about pentosans content in *Triticum monococcum* is available in the literature. Interestingly, when pentosans content in flour increases, the retrogradation of starch decreases, due to their steric interference with the intermolecular associations of starch [43].

A peculiar characteristic of einkorn flour is its yellowness, due to the abundant presence of carotenoid [4,5]. This is confirmed by the yellow pigment content of both ID331 (8.72 mg/kg DM) and Monlis (11.30 mg/kg DM), which are about ten times larger than the value of Blasco (0.98 mg/kg DM) and in the range reported for whole meal flours of a collection of einkorns [1]. Remarkably, the two einkorns tested had concentrations that were twice higher than those commonly reported for yellow durum wheats [44].

The two-way ANOVAs (Table S2) verified the existence of highly significant differences among genotypes, environments, and their interactions for dry gluten content, Falling number, α-amylase activity, and SDS sedimentation value; the genotypic effect was largely the most relevant factor. The results of the four environments, not discussed in this article, are summarized in Table S3 for completeness of information.

As already hinted by the protein content, the dry gluten content was higher in ID331 and Monlis than in Blasco (Table 1). Falling number and α-amylase activity are inversely correlated traits [45]: the higher the α-amylase activity, the shorter the falling time. In this study, Falling Number results were all well above those values suggesting modest pre-germination phenomena (200–300 s) [46]. The significantly higher levels of both FN and α-amylase activities obtained for einkorn flours can be explained with their superior ash content, being that the amylolytic enzymes are mainly localized in the germ and in the external regions of the kernels.

The SDS sedimentation test underlined the good breadmaking propension of the two einkorns tested and of the bread wheat control. Einkorn generally has a poor breadmaking capacity [32,33], but some good accessions are reported [1,6,7,47]. Indeed, ID331 and Monlis are among the most suitable *T. monococcum* to prepare leavened products, and their SDS quality was superior even to the good breadmaking wheat variety Blasco.

The farinographic results (Figure S1) suggested differences between the bread wheat flour and the two einkorn flours only for water absorption (Table 2), as Blasco needed more water (around 6%) than ID331 and Monlis to reach optimum dough consistency. Development time, stability, and degree of softening did not look different. Borghi et al. [6] found that most of the einkorn accessions that they investigated had very low stability (<1 min), but some genotypes reached 2.0–4.5 min. Low development time, poor stability, and a strong degree of softening are indices of a weak flour, with scarce resistance to the mechanical action of kneading and are therefore unsuitable for preparing leavened products. Nevertheless, ID331 and Monlis showed a breadmaking attitude similar to Blasco, a cultivar fit for the manufacturing of high-quality leavened bakery products.

The unreplicated results of the rheofermentographic parameters are summarized in Table 2 and are presented across the four locations in Figure S2. This analysis is helpful to evaluate changes in dough during the leavening phase that can be linked to breadmaking quality [29]. The results in Figure S2 suggest that Monlis and ID331 reached a significantly higher dough development during the test in a shorter time in comparison to Blasco, maybe due to the smaller size of flour (see above) and starch granules [48], whose superior surface-to-volume ratio may favor enzymatic reactions. They also exhibited a limited CO_2_ loss, which determined a significantly higher CO_2_ retention coefficient, probably attributable to a more compact gluten network due to the higher protein content of the two einkorn flours.

The two-way ANOVA (Table S2) of the viscoelasticity parameters hinted to significant differences between Blasco and the two einkorns, both for the limits of the linear viscoelastic region obtained from the strain sweep test, and the storage and loss moduli obtained from the frequency sweep test. For completeness of information, the results of the four environments, not discussed in this article, are summarized in Table S3.

The evaluation of the region of linear viscoelasticity of a sample is an important step: when materials are tested in the linear range, their viscoelastic behaviors do not depend on the magnitude of the stress, the magnitude of the deforming strain, or the rate of application of the strain [49], but on their intrinsic features. The length of the linear viscoelastic region (LVR) can therefore be used as a measurement of dough stability: einkorn doughs remained in the linear viscoelastic region over greater strains than Blasco dough, indicating the presence of a stronger network. Indeed, for Blasco a drop in G′ LVR started to occur above the 0.33% strain and became larger at a higher strain, indicating a progressive disorganization of the dough structure beyond this deformation level. A 0.1% strain for the subsequent frequency tests was therefore adopted, as within the LVR of all the samples. Although all the dough samples that were analyzed had the same farinographic consistency (500 BU), significant differences were observed among the samples. Generally, the mechanical spectra of all the samples exhibited a solid-like behavior, with G′ always being higher than G″. Einkorn doughs presented significantly higher values of both moduli, with a prevalence of the viscous behavior, as indicated by the higher values of the damping factor (G″/G′).

### 3.3. Bread

#### 3.3.1. Characteristics

The two-way ANOVA for bread volume, height, and specific volume (Table S2) scored significant genetic effects, including especially highly significant environmental effects and their interactions. Interestingly, ID331 and Monlis outcompeted Blasco for loaf volume, height, and specific volume (Table 2), indicating that it is possible to have an einkorn flour with the same technological properties of good quality bread wheat. Cross-sections of the loaves prepared with the flours from the LENObio trial are depicted in Figure 1.

**Figure 2:**
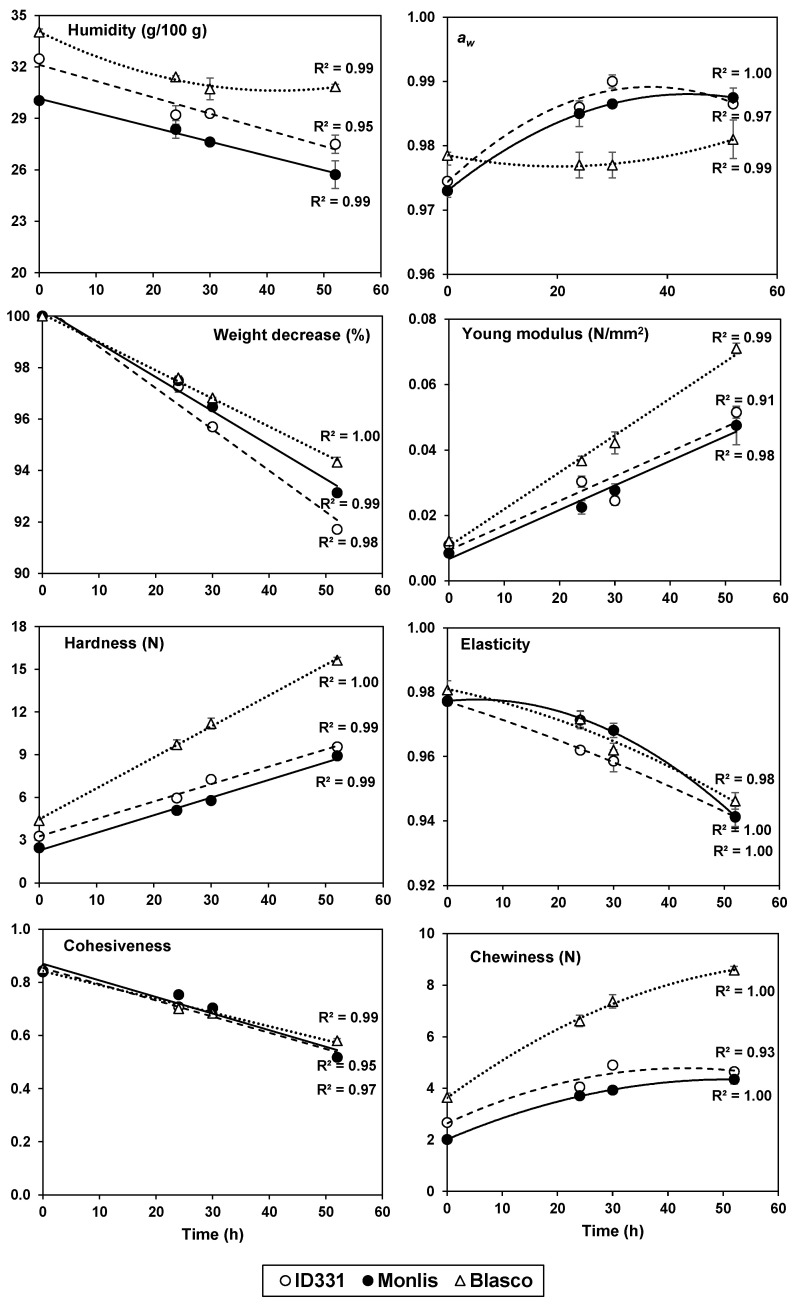
Moisture, water activity (a_w_), weight decrease, and texture parameters of breads from two einkorns (ID331 and Monlis) and one bread wheat (Blasco) during storage (h). Error bars represent standard error.

Due to the higher amount of water added following indication of the farinographic test (56.0% vs. 53.4% to reach 500BU), the bread produced from Blasco flour had a significantly higher loaf weight (Table 2), crumb humidity, and a_w_ (Figure 2, t_0_) than the einkorn samples. Monlis exhibited the highest loaf volume (on average, 740 cm^3^), followed by ID331 (732 cm^3^) and Blasco (671 cm^3^). Similar values for loaves obtained from selected einkorn flours are reported [6,7]. These results were reflected in the breads’ specific volumes: Monlis originated the breads with the highest value, that is the “lightest” breads (Table 2).

The one-way ANOVA (Table S4) indicated the presence of significant differences among genotypes for color indices in the cases of a* (crust and crumb) and b* (crumb). Table 3 reports the average values of L*, a*, and b* evaluated on the crust and crumb of each bread sample. The crust color showed a low variation, with very similar L*, a*, and b* values among samples, as observed also by D’Egidio et al. [31]. A difference was instead evident for crumb color: Blasco had significantly higher a* values, but a much inferior b* (yellow index) than both the einkorn genotypes. This reflects the differences in the yellow pigment content of the raw materials (Table 1), which was particularly abundant in the carotenoid-rich einkorns and gives their breads a characteristic and enticing golden yellow color. Table 3 also shows the average values of the color parameters evaluated by Image Analysis; the ANOVA (Table S4) highlighted significant differences among the samples for all parameters: Blasco had significantly lower R (red intensity) and G (green intensity), and had significantly higher B (blue intensity) than the einkorn samples.

Bread can be considered a solid food foam because the gas developed during fermentation is trapped in its solid matrix. Information about shape, size, and distribution of the pores is very important in order to identify the influence of processing conditions on the quality of the final products and to devise methods to manufacture products with peculiar textural characteristics [50]. Image Analysis was used in this study to assess the porosity of the different breads by determining the number of pores and their geometrical features (area, diameter, shape). All the identified pores were classified according to their size and the results are shown in Table 4, while the ANOVA is presented in Table S5. The Blasco breads presented as many small pores (0.1–1 mm^2^) as the einkorn breads, while the number of medium-sized pores (1–5 mm^2^) was significantly higher (*p* ≤ 0.05). Among einkorns, Monlis bread porosity was characterized by a greater number of large pores (>5 mm^2^), with an average area of 9.21 mm^2^, significantly higher than the Blasco and ID331 pores of the same class.

Information about the shape and regularity of the pores is obtained by considering that the larger the value, the greater the irregularity. Indeed, the smallest pores had the most regular and uniform shape (range: 1.78–1.89), while the large pores ranged from 2.34 to 2.60, indicating that as their size increased, their form became more irregular and less homogeneous. In our case, ID331 bread had the most regular pores for all the three size classes: its bread presented a porosity similar to Blasco, but was characterized by a more regular shape, thus making the porosity of the crumb more homogeneous.

The number of pores and their average area is reflected in the percentage of relative area occupied in the slice and, ultimately, in the total porosity area of the bread. For Blasco, the surface occupied by the small pores was similar to that found in the einkorn samples, while the surface associated with medium-sized pores was significantly higher. Overall, pores area was highest in Blasco bread (810.57 mm^2^), mainly because of the greater number of medium-sized pores. In Monlis bread, the large pores contributed considerably to the total porosity, as also confirmed by the less homogeneous internal loaf structure. Nevertheless, the loaves obtained from Monlis were the ones with the largest specific volumes, probably due to the high presence of these large pores. Despite the distribution of pores in the three classes being different among the three samples, the percentage of pores, computed as ratio between total alveolate area and total bread slice area, was similar among the three accessions.

The mechanical and geometrical characteristics of bread influence its behavior during oral processing [50]. To assess these features in a reproducible way, Texture Profile Analysis is used to create controlled stresses and to measure the mechanical characteristics of foods. Figure 2 shows the average values of the texture parameters of the three different breads. The two-way ANOVA (Table S6) stressed the existence of significant differences (*p* ≤ 0.05) between accessions only for young modulus (consistency index), hardness, gumminess, and chewiness. Storage times were always significant and so was the genotype x time interaction (except for elasticity). At t_0_, Blasco showed the highest consistency, hardness, gumminess, and chewiness, and Monlis exhibited the lowest values, while in general ID331 had results in line with Monlis. These data indicate how the bread loaves obtained from Monlis were the softest ones and were characterized by the best chewability, despite the finer and more regular porosity and the higher moisture content of Blasco breads, which are factors that are conventionally related to a softer structure. Possibly, the superior softness of the einkorn breads may be related to the lower starch content of their flours.

#### 3.3.2. Changes during Storage

Figure 2 also shows the evolution of a_w_, weight, young modulus, and texture parameters of the breads’ slices during storage. Blasco bread lost most of its moisture in the first 24 h after the production, while the decrease was more progressive for the einkorns. Interestingly, Blasco bread did not present significant water activity changes during storage, while the einkorn breads showed a significant increase after the first 24 h, followed by a plateau. This increase could be due to a reorganization of starch molecules to a different composition of starch with particular reference to the amylose content whose molecules, involved in the phenomenon of retrogradation, have a greater tendency to associate with each other and form hydrogen bonds, releasing water, or even to a reorganization of the protein matrix [51], more abundant in einkorn flours. As expected, the weight of all the breads significantly decreased during storage, but was steeper for the two einkorns after the initial 24 h.

The Young modulus of the Blasco bread at t_0_ was higher than that of the einkorn breads. During storage, this index increased for all samples, but was faster in the bread wheat. This behavior is also reflected in the hardness: in fact, the loaves from einkorn flours showed, already at t_0_, lower hardness values (ID331: 3.26 N; Monlis: 2.44 N) compared with Blasco (4.35 N), and this difference amplified over time, as the kinetics of the bread from bread wheat were more accelerated than those of einkorn breads. The speed of the process, as measured by the value of the angular coefficient of the regression curves, was 0.24 N/h for Blasco and 0.12–0.14 N/h for the two einkorn samples. Hence, the einkorn breads maintained their softness for a longer time and their retrogradation was slower than that of the bread wheat.

Elasticity and cohesiveness decreased with similar trends in einkorn and wheat breads over the storage time. For that reason, bread chewiness (i.e., the energy required to chew a solid food) was mainly related to the hardness values, which was much higher for wheat bread, particularly during storage. Since the superior softness of the einkorn breads cannot be attributed to a higher moisture content (indeed, Blasco breads were characterized by the highest moisture), such behavior may be related to the lower starch content of einkorn flours and/or to the lower presence of amylose in the samples. Additionally, the slightly higher amylase activity of Monlis and ID331 may have contributed because amylases decrease starch retrogradation, diminish rigidity of the starch gel network, and limit starch–protein interactions [52,53]. Their anti-staling effect is also related to the hydrolyzation of amylopectin and the production of soluble low-molecular-weight branched-chain polymers, which are less prone to retrogradation and influence water movement and accessibility [54].

## 4. Conclusions

This study demonstrates the possibility of obtaining einkorn breads with technological properties comparable to those of breadmaking-quality bread wheat. However, to achieve this result, high-quality genotypes are necessary. Furthermore, as the quality characteristics of the breads are influenced by processing conditions, einkorn bread manufacturing should utilize short mixing and leavening times to avoid overstressing the doughs. The choice of appropriate einkorn varieties and optimization of the breadmaking process will allow the production of breads with appropriate technological characteristics, endowing the market with an innovative cereal-based food possessing a superior nutritional value and longer shelf life.

## Figures and Tables

**Table 1:** Kernel and flour parameters (mean values ± standard error) of two einkorns (ID331 and Monlis) and one bread wheat (Blasco). *n* = 8: four environments × two repetitions.

	ID331	MONLIS	BLASCO
Kernel *			
Hectolitre weight (kg/hL)	76.6 ± 1.1	75.8 ± 1.4	85.3 ± 1.4
Flour yield (g/100 g)	61.8 ± 0.7	59.8 ± 1.1	54.0 ± 2.5
Flour <125 μm (g/100 g)	85.3 ±1.6	86.6 ± 1.8	57.8 ± 2.9
**Flour**			
Moisture (g/100 g)	14.5 ±0.4	14.3 ± 0.4	14.5 ± 0.3
Ash (g/100 g DM)	0.60 ^b^ ± 0.02	0.67 ^a^ ± 0.01	0.59 ^c^ ± 0.01
Protein (g/100 g DM)	16.7 ^a^ ± 1.5	16.2 ^b^ ± 1.3	10.5 ^c^ ± 0.8
Starch (g/100 g DM)	69.3 ^b^ ± 1.3	68.7 ^b^ ± 1.8	79.1 ^a^ ± 1.1
Amylose (g/100 g starch)	25.3 ^b^ ± 0.1	26.9 ^a^ ± 0.3	26.8 ^a^ ± 0.3
Total pentosans (g/100 g DM)	2.49 ± 0.01	2.66 ± 0.08	2.52 ± 0.05
Soluble pentosans (g/100 g DM)	0.95 ^b^ ± 0.04	1.10 ^a^ ± 0.03	0.82 ^c^ ± 0.01
Yellow pigment (mg/kg DM)	8.7 ^b^ ± 0.6	11.3 ^a^ ± 0.7	1.0 ^c^ ± 0.1
Dry gluten (g/100 g DM)	1.58 ^a^ ±0.13	1.49 ^a^ ± 0.08	1.15 ^b^ ± 0.03
Falling number (s)	358 ^b^ ± 16	365 ^b^ ± 11	431 ^a^ ± 13
α-amylase activity (CU/g DM)	0.19 ^a^ ± 0.02	0.20 ^a^ ± 0.01	0.15 ^b^ ± 0.01

Different letters in the same row mean significant differences (*p* ≤ 0.05) among samples following LSD test. * The kernel data were not subjected to statistical analysis.

**Table 2:** Breadmaking parameters (mean values ± standard error) of two einkorns flours (ID331 and Monlis) and one bread wheat flour (Blasco). *n* = 8: four environments × two repetitions (Brabender, *n* = 4: four environments).

	ID331	MONLIS	BLASCO
SDS sedimentation volume (mL)	91 ^a^ ± 0.9	88 ^a^ ± 2	66 ^b^ ± 7
Brabender farinograph *			
Water absorption (%)	52.7 ± 1.5	52.4 ± 0.8	58.8 ± 0.6
Development time (s)	144 ± 15	168 ± 23	195 ± 107
Stability time (s)	387 ± 226	305 ± 122	429 ± 234
Degree of softening (BU)	65 ± 20	85 ± 15	56 ± 22
Rheofermentograph *			
Dough max height (mm)	72.3 ± 5.3	73.8 ± 4.8	63.0 ± 3.6
Time to max height (min)	121.8 ± 10.8	119.8 ± 20.5	175.0 ± 4.4
CO_2_ total (mL)	1136 ± 89	1168 ± 107	1713 ± 79
CO_2_ lost (mL)	12 ± 5	15 ± 5	193 ± 26
CO_2_ retained (mL)	1124 ± 85	1154 ±103	1523 ± 85
Retention coefficient (%)	99.0 ± 0.4	99.3 ± 0.1	88.7 ± 1.6
**Strain sweep test**			
LVR limit for G′ (%)	0.70 ^a^ ± 0.03	0.69 ^a^ ± 0.02	0.33 ^b^ ± 0.02
LVR limit for G″ (%)	0.82 ^a^ ± 0.03	0.79 ^a^ ± 0.01	0.48 ^b^ ± 0.02
**Frequency sweep test**			
G′ (Pa) (0.10% strain, 1 Hz)	9216 ^b^ ± 822	10,748 ^a^ ± 860	7764 ^c^ ± 605
G″ (Pa) (0.10% strain, 1 Hz)	4403 ^b^ ± 270	5131 ^a^ ± 565	2933 ^c^ ± 151
Damping factor G″/G′	0.48 ^a^ ± 0.02	0.48 ^a^ ± 0.02	0.38 ^b^ ± 0.02
**Bread**			
Volume (cm^3^)	732 ^a^ ± 128	740 ^a^ ± 107	671 ^b^ ± 43
Height (cm)	102 ^ab^ ± 12	104 ^a^ ± 10	99 ^b^ ± 3
Specific volume (cm^3^/kg)	5.23 ^ab^ ± 0.97	5.40 ^a^ ± 0.77	4.55 ^b^ ± 0.29

Different letters in the same row mean significant differences (*p* ≤ 0.05) among samples following LSD test. * The farinograph and rheofermentograph data were not subjected to statistical analysis.

**Table 3:** Crust and crumb colorimetric indices (mean values ± standard error) of breads prepared from flours of two einkorns (ID331 and Monlis) and one bread wheat (Blasco) cropped at Sant’Angelo Lodigiano under conventional management. *n* = 12: three sections from two slices of two loaves.

		ID331	Monlis	Blasco
Crust	L*	46.1 ± 3.1	45.7 ± 2.6	46.1 ± 2.7
	a*	17.3 ^ab^ ± 0.5	17.0 ^b^ ± 0.4	17.7 ^a^ ± 0.7
	b*	26.7 ± 2.7	26.8 ± 2.2	25.4 ± 1.5
Crumb	L*	79.6 ± 0.9	79.2 ± 0.5	79.6 ± 1.8
	a*	−2.6 ^b^ ± 0.1	−2.9 ^c^ ± 0.1	0.2 ^a^ ± 0.08
	b*	35.2 ^a^ ± 0.6	35.8 ^a^ ± 0.2	15.5 ^b^ ± 0.4
	*R*	234 ^a^ ± 5	232 ^a^ ± 7	219 ^b^ ± 4
	*G*	216 ^a^ ± 5	214 ^a^ ± 7	207 ^b^ ± 3
	*B*	130 ^b^ ± 3	126 ^c^ ± 4	172 ^a^ ± 3

Different letters in the same row mean significant differences (*p* ≤ 0.05) among samples following LSD test.

**Table 4:** Porosity (mean values ± standard error) of the breads prepared from two einkorn (ID331 and Monlis) and one bread wheat (Blasco) refined flours cropped at Sant’Angelo Lodigiano under conventional management. *n* = 12: three sections from two slices of two loaves. Porosity classes: C1 = 0.1–1 mm^2^, C2 = 1–5 mm^2^, C3 = >5 mm^2^. Different letters within pores class in the same column indicate significant differences (*p* ≤ 0.05) among samples following LSD test.

		N° Pores	N° Pores (%)	Pores Area (mm^2^)	Pores Area (%)	Mean Pores Area (mm^2^)	Mean Pores Diametre (mm)	Pores Shape
C1	ID331	647 ± 34	75.7 ± 2.8	237.5 ^a^ ± 13.1	32.6 ± 3.6	0.37 ± 0.01	0.64 ^b^ ± 0.01	1.78 ± 0.03
	Monlis	611 ± 30	73.9 ± 2.8	219.6 ^b^ ± 11.4	27.9 ± 4.3	0.36 ± 0.01	0.64 ^b^ ± 0.01	1.89 ± 0.05
	Blasco	652 ± 32	72.7 ± 1.6	243.5 ^a^ ± 12.6	30.1 ± 2.2	0.37 ± 0.01	0.65 ^a^ ± 0.01	1.89 ± 0.04
C2	ID331	193 ^b^ ± 32	22.5 ± 2.9	377.8 ^b^ ± 63.2	51.2 ^b^ ± 4.5	1.96 ± 0.08	1.55 ± 0.04	1.98 ^b^ ± 0.05
	Monlis	195 ^b^ ± 23	23.6 ± 2.2	388.0 ^b^ ± 57.2	48.5 ^b^ ± 0.8	1.98 ± 0.08	1.57 ± 0.03	2.16 ^a^ ± 0.08
	Blasco	229 ^a^ ± 14	25.6 ± 1.2	454.9 ^a^ ± 31.7	56.2 ^a^ ± 2.3	1.98 ± 0.05	1.58 ± 0.02	2.12 ^a^ ± 0.09
C3	ID331	15 ^b^ ± 3	1.8 ^b^ ± 0.5	118.1 ^b^ ± 28.1	16.1 ^b^ ± 3.6	7.97 ^b^ ± 0.59	2.93 ± 0.16	2.34 ± 0.34
	Monlis	21 ^a^ ± 6	2.5 ^a^ ± 0.7	191.4 ^a^ ± 51.0	23.6 ^a^ ± 3.9	9.21 ^a^ ± 0.94	3.09 ± 0.18	2.39 ± 0.38
	Blasco	15 ^b^ ± 4	1.7 ^b^ ± 0.4	112.2 ^b^ ± 35.5	13.7 ^b^ ± 3.9	7.37 ^b^ ± 0.95	2.86 ± 0.13	2.60 ± 0.36

## Data Availability

The data presented in this study are available in the article and the Supplementary Materials.

## Supplementary Materials

The following supporting information can be downloaded at: https://www.mdpi.com/article/10.3390/foods12081610/s1, Figure S1: Hectoliter weight, flour yield, and proportion of flour <125 μm of flours of ID331, Monlis, and Blasco cropped in four locations (SAL, SALbio, LENObio, and ROMA); Figure S2: Reofermentographic results of flours of ID331, Monlis, and Blasco cropped in four locations (SAL, SALbio, LENObio, and ROMA). Table S1: Cropping environments and field management information; Table S2: Two-way ANOVA (* *p* ≤ 0.05; ** *p* ≤ 0.01; *** *p* ≤ 0.001) of composition, rheological analysis, and breadmaking test parameters; Table S3: Environmental values (mean ± s.e.) of flour and bread parameters of two einkorns (ID331 and Monlis) and one bread wheat (Blasco); Table S4: One-way ANOVA (* *p* ≤ 0.05; ** *p* ≤ 0.01; *** *p* ≤ 0.001) of crust and crumb color parameters; Table S5. One-way ANOVA (* *p* ≤ 0.05; ** *p* ≤ 0.01; *** *p* ≤ 0.001) of porosity; Table S6: Two-way ANOVA (* *p* ≤ 0.05; ** *p* ≤ 0.01; *** *p* ≤ 0.001) of texture profile analysis parameters.
