# Supplementary material for: Breadmaking Performance of Elite Einkorn (Triticum monococcum L. subsp. monococcum) Lines: Evaluation of Flour, Dough and Bread Characteristics
Source: Foods. 2023 Apr 10;12(8):1610. doi: 10.3390/foods12081610 (PMC10137832; doi:10.3390/foods12081610)
Supplement: Supplementary file 1 [file foods-12-01610-s001.zip › Figure S1.pdf]

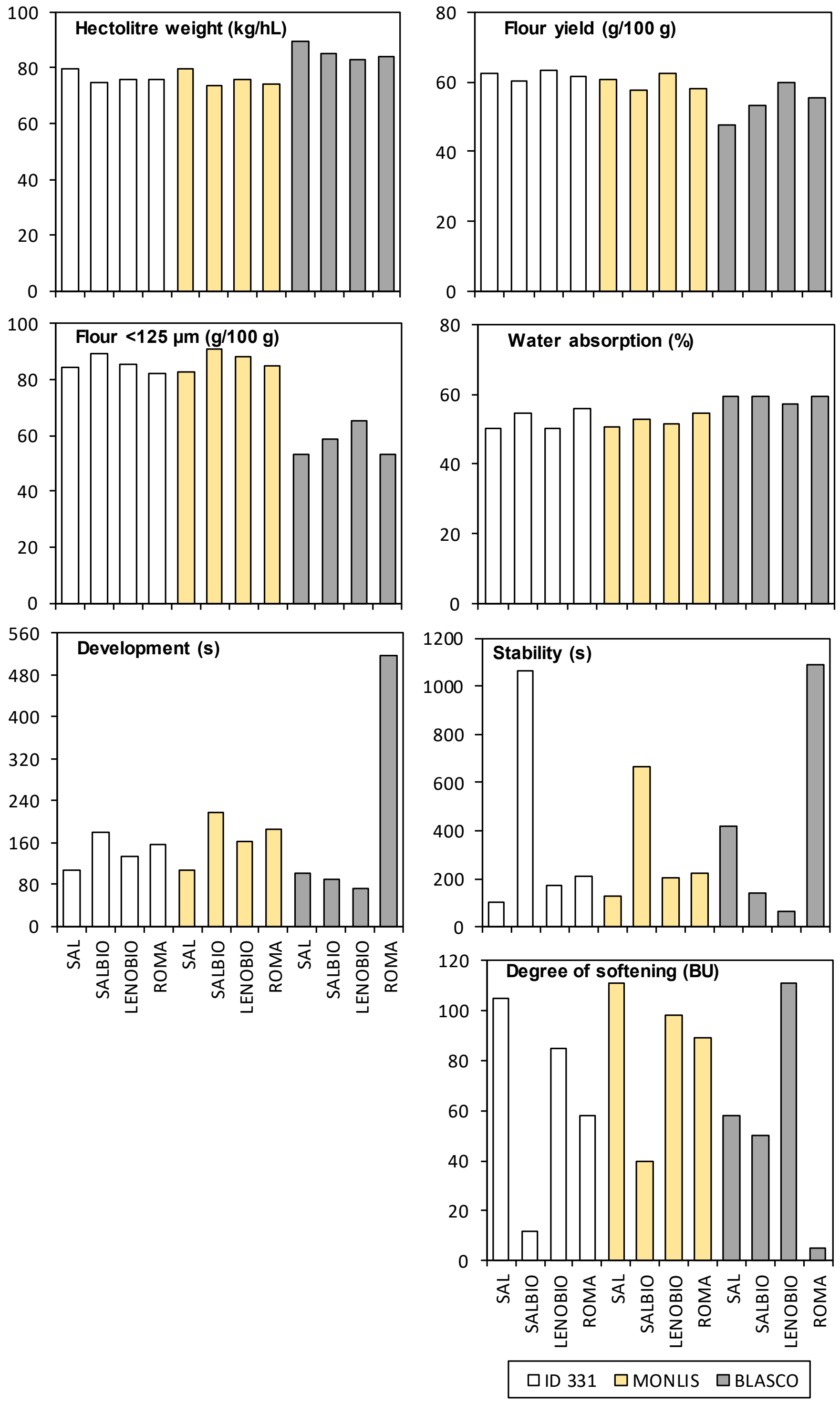

Figure S1. Hectolitre weight, flour yield and proportion of flour < 125 µm of flours of ID331, Monlis and Blasco cropped in four locations (SAL, SALbio, LENObio and ROMA).
