# Supplementary material for: Breadmaking Performance of Elite Einkorn (Triticum monococcum L. subsp. monococcum) Lines: Evaluation of Flour, Dough and Bread Characteristics
Source: Foods. 2023 Apr 10;12(8):1610. doi: 10.3390/foods12081610 (PMC10137832; doi:10.3390/foods12081610)
Supplement: Supplementary file 1 [file foods-12-01610-s001.zip › Table S1 environment new.pdf]

Table S1. Cropping environments and field management information.

	SAL	SALbio	LENObio	Rome
Management	Conventional	Organic	Organic	Conventional
Soil	Sandy	Sandy	Sandy-loamy	Sandy-loamy
Previous crop	Maize	Pea	Barley	Broad bean
Nitrogen (kg/ha)	40*	80+	80+	100*
Harvesting date	17/7	15/7	16/7	11/7

* ammonium nitrate (26% N; Yara Italia, Milan, Italy).

+ Endurance N8 (8% N; Unimer, Vidor, TV, Italy), an organic nitrogen fertilizer. The organic nitrogen, from protein sources, derives from meat meal with a significant blood content. The meat meal is characterized by the presence of amino acids such as glycine, proline, alanine, glutamic acid and hydroxyproline, in addition to calcium phosphate. These elements are gradually made available to the crop by the microfauna present in the dried manure component. The blood, present in the mixture, contains proteins at their native state, a highly bioavailable source of organic nitrogen for the activity of microorganisms in the soil; in addition provides 0.5% iron (as iron sulphate) and 0.01% zinc (as zinc sulphate).

Temperature and rainfall of Sant'Angelo Lodigiano (SAL and SALbio), Leno (LENObio) and ROMA

	Mean Temperature (°C)			Rainfall (mm)		
	SAL/SALbio	LENObio	ROMA	SAL/SALbio	LENObio	Roma
April	13.8	11.8	13.7	56.2	38.8	66.8
May	18.8	16.4	17.8	12.6	83.2	44.7
June	23.6	20.2	21.2	2.4	26.6	15.5
July*	26.2	23.2	25.1	*2.5*	16.2	27.1

*Up to July 14
