# Supplementary material for: Breadmaking Performance of Elite Einkorn (Triticum monococcum L. subsp. monococcum) Lines: Evaluation of Flour, Dough and Bread Characteristics
Source: Foods. 2023 Apr 10;12(8):1610. doi: 10.3390/foods12081610 (PMC10137832; doi:10.3390/foods12081610)
Supplement: Supplementary file 1 [file foods-12-01610-s001.zip › Table S2 flour ANOVA new.pdf]

Table S2. Two-way ANOVA results (* $p \leq 0.05$; ** $p \leq 0.01$; *** $p \leq 0.001$) of composition, rheological analysis and breadmaking test parameters.

VARIABLE	GENOTYPE (G)	ENVIRONMENT (E)	GxE	ERROR
d.f.	*2*	*3*	*6*	*12*
Moisture	0.057***	0.218***	0.379***	0.001
Ash	0.012***	0.003***	0.002***	0.0002
Protein	95.7***	26.1***	5.0***	0.1
Total pentosans	6.86***	2.06***	0.95*	0.21
Soluble pentosans	0.695***	0.150***	0.007	0.020
Starch	196.7***	12.3*	13.1*	3.0
Amylose	6.42***	0.42	0.53	0.53
Yellow pigment	230.6***	4.4***	1.3***	0.1
Dry gluten	0.411***	0.124***	0.032*	0.008
Falling number	12900***	1659***	1450***	129
α-amylase	0.0054***	0.0019***	0.0011***	0.0001
SDS sedimentation volume	1512.8***	95.6**	146.7***	10.1
**Rheological analysis**				
Strain sweep test:				
Deformation limit G'	0.347***	0.010***	0.002	0.001
Deformation limit G''	0.279***	0.006	0.002	0.002
Frequency sweep test:				
Storage modulus G'	17809700***	5869160***	4195290***	509008
Loss modulus G''	10035300***	1473740***	921188***	101688
$\tan\delta = G''/G'$	0.0256***	0.0022***	0.0027***	0.0001
**Breadmaking test**				
Bread volume	24448***	117306***	56045***	164
Bread height	182.4***	1155.8***	415.5***	5.6
