# Supplementary material for: Breadmaking Performance of Elite Einkorn (Triticum monococcum L. subsp. monococcum) Lines: Evaluation of Flour, Dough and Bread Characteristics
Source: Foods. 2023 Apr 10;12(8):1610. doi: 10.3390/foods12081610 (PMC10137832; doi:10.3390/foods12081610)
Supplement: Supplementary file 1 [file foods-12-01610-s001.zip › Table S3 environment means new.pdf]

Table S3. Environmental values (mean ± s.e.) of flour and bread parameters of two einkorns (ID331 and Monlis) and one bread wheat (Blasco). N=6: three accessions and two repetitions.

	SAL	SALbio	LENObio	ROMA
**Flour**				
Moisture (g/100 g)	14.2±0.2	14.5±0.2	14.3±0.2	14.6±0.1
Ash (g /100 g)	0.62±0.01	0.64±0.017	0.60±0.018	0.59±0.021
Protein (g/100 g)	12.6±0.6	16.3±1.9	12.8±1.4	16.3±1.2
Starch (g/100 g)	63.8±1.2	61.1±2.7	62.2±1.9	60.6±2.2
Amylose (g/100 g starch)	26.4±0.3	26.1±0.3	26.2±0.4	26.7±0.6
Total pentosans (g/100 g)	6.2±0.3	7.4±0.5	6.4±0.5	7.2±0.3
Soluble pentosans (g/100 g)	1.51±0.11	1.87±0.12	1.60±0.11	1.58±0.13
Yellow pigment (mg/kg)	6.2±1.7	8.1±2.3	7.3±2.1	6.4±1.8
Dry gluten (g/100 g)	1.28±0.03	1.56±0.14	1.29±0.08	1.50±0.10
Falling number (s)	365.7±11.0	392.3±21.7	403.7±6.8	377.7±23.7
α-amylase activity (CU/g flour)	0.20±0.01	0.19±0.015	0.16±0.007	0.17±0.004
SDS sedimentation volume (mL)	83.7±3.0	81.0±5.4	76.8±9.2	86.2±3.4
**Strain sweep test**				
Deformazione limite G' (%)	0.55±0.06	0.60±0.08	0.53±0.08	0.62±0.08
Deformazione limite G" (%)	0.74±0.08	0.67±0.06	0.69±0.08	0.69±0.06
**Frequency sweep test**				
Storage modulus G' (Pa)	8891±719	10280±967	9765±799	8033±374
Loss modulus G" (Pa)	3803±324	4802±656	4283±522	3728±293
Damping factor (G''/G')	0.43±0.01	0.46±0.04	0.43±0.02	0.46±0.02
**Bread**				
Volume (cm^3)	592±41	920±130	675±40	671±57
Height (cm)	90±5	121±10	100±3	95±5
Weight (g)	143±3	141±3	141±2	142±3
Specific volume (cm^3/g)	4.16±0.23	6.59±0.76	4.78±0.22	4.71±0.24
