# Supplementary material for: Breadmaking Performance of Elite Einkorn (Triticum monococcum L. subsp. monococcum) Lines: Evaluation of Flour, Dough and Bread Characteristics
Source: Foods. 2023 Apr 10;12(8):1610. doi: 10.3390/foods12081610 (PMC10137832; doi:10.3390/foods12081610)
Supplement: Supplementary file 1 [file foods-12-01610-s001.zip › Table S4 colour ANOVA new.pdf]

Table S4. One-way ANOVA results (* $p \leq 0.05$; ** $p \leq 0.01$; *** $p \leq 0.001$) of crust and crumb colour parameters determined on the breads prepared from the SAL flours.

VARIABLE	GENOTYPE	ERROR
d.f.	*2*	*21*
**Crust**		
*L**	0.53	6.88
*a**	0.90*	0.21
*B**	4.77	4.14
**Crumb**		
*L**	0.26	1.45
*a**	11.48***	0.01
*B**	535.60***	0.21
**Image Analysis**		
R	405.19***	26.15
G	115.66*	27.85
B	3966.58***	11.64
