# Supplementary material for: Breadmaking Performance of Elite Einkorn (Triticum monococcum L. subsp. monococcum) Lines: Evaluation of Flour, Dough and Bread Characteristics
Source: Foods. 2023 Apr 10;12(8):1610. doi: 10.3390/foods12081610 (PMC10137832; doi:10.3390/foods12081610)
Supplement: Supplementary file 1 [file foods-12-01610-s001.zip › Table S5 pores ANOVA new.pdf]

Table S5. One-way ANOVA results (* $p \leq 0.05$; ** $p \leq 0.01$; *** $p \leq 0.001$) of porosity determined on the breads prepared from the SAL flours.

VARIABLE	GENOTYPE	ERROR
d.f.	*2*	*15*
**C1**		
N° pores	3048.7	1029.4
N° pores %	14.0	6.1
Pores area	927.1*	153.7
Pores area %	33.3	12.2
Mean pores area	0.00033	0.00016
Mean pores diametre	0.00061**	0.00008
Pores shape	0.0235***	0.0017
**C2**		
N° pores	2471.1*	601.9
N° pores %	14.8	4.89
Pores area	10530.3*	2754.5
Pores area %	91.3***	8.6
Mean pores area	0.00127	0.00550
Mean pores diametre	0.00137	0.00093
Pores shape	0.05796**	0.00550
**C3**		
N° pores	74.1*	21.0
N° pores %	1.3*	0.3
Pores area	11680.1**	1551.2
Pores area %	159.8**	14.3
Mean pores area	5.31	0.71
Mean pores diametre	0.08303	0.02472
Pores shape	0.11577	0.13173

Porosity classes: C1 = 0.1-1 mm^2, C2 = 1-5 mm^2, C3 = >5 mm^2.
