# Supplementary material for: Breadmaking Performance of Elite Einkorn (Triticum monococcum L. subsp. monococcum) Lines: Evaluation of Flour, Dough and Bread Characteristics
Source: Foods. 2023 Apr 10;12(8):1610. doi: 10.3390/foods12081610 (PMC10137832; doi:10.3390/foods12081610)
Supplement: Supplementary file 1 [file foods-12-01610-s001.zip › Table S6- Texture ANOVA new.pdf]

Table S6. Two-way ANOVA results (* p ≤ 0.05; ** p ≤ 0.01; *** p ≤ 0.001) of texture profile analysis parameters.

VARIABLE		GENOTYPE (G)	TIME (E)	GxT	ERROR
	d.f.	*2*	*3*	*6*	*59*
Young modulus		0.00129***	0.00628***	0.00016***	0.00003
Hardness		146.24***	185.46***	10.38***	0.031
Elasticity		0.00015	0.00416***	0.00011	0.00006
Cohesiveness		0.00091	0.27224***	0.00400***	0.00031
Gumminess		68.08***	36.76***	3.76***	0.15
Chewiness		63.15***	31.51***	3.37***	0.14
